# Efficacy of regional anesthesia in hip preservation surgeries: a systematic review

**DOI:** 10.1093/jhps/hnad008

**Published:** 2023-04-15

**Authors:** Evan M Banks, Jake A Ayisi, Aliya G Feroe, Walid Alrayashi, Yi-Meng Yen, Eduardo N Novais, Mahad M Hassan

**Affiliations:** Department of Orthopaedic Surgery, University of Minnesota Medical School, 2450 Riverside Ave Suite R200, Minneapolis, MN 55454, USA; Boston University Graduate Medical Sciences, Boston University School of Medicine, 72 East Concord St., L-317, L309, Boston, MA 02118, USA; Department of Orthopaedic Surgery, Harvard Medical School, 25 Shattuck St, Boston, MA 02115, USA; Department of Anesthesiology, Critical Care, and Pain Medicine, Boston Children’s Hospital and Harvard Medical School, 300 Longwood Ave, Boston, MA 02115, USA; Department of Orthopaedic Surgery, Child and Young Adult Hip Preservation Program, Boston Children’s Hospital and Harvard Medical School, 300 Longwood Avenue, Boston, MA 02115, USA; Department of Orthopaedic Surgery, Child and Young Adult Hip Preservation Program, Boston Children’s Hospital and Harvard Medical School, 300 Longwood Avenue, Boston, MA 02115, USA; Department of Orthopaedic Surgery, University of Minnesota Medical School, 2450 Riverside Ave Suite R200, Minneapolis, MN 55454, USA; Tria Orthopedic Center, 8100 Northland Dr., Bloomington, Minneapolis, MN 55431, USA

## Abstract

The purpose of this study was to review the current literature on perioperative pain management in hip arthroscopy, periacetabular osteotomy and surgical hip dislocation. A systematic review of the literature published from January 2000 to December 2022 was performed. Selection criteria included published randomized controlled trials, prospective reviews and retrospective reviews of all human subjects undergoing hip preservation surgery. Exclusion criteria included case reports, animal studies and studies not reporting perioperative pain control protocols. Thirty-four studies included hip arthroscopy in which peripheral nerve blocks were associated with a significant reduction in pain score (*P* = 0.037) compared with general anesthesia alone. However, no pain control modality was associated with a significant difference in postanesthesia care unit opioid use (*P* = 0.127) or length of stay (*P* = 0.251) compared with general anesthesia alone. Falls were the most common complication reported, accounting for 37% of all complications. Five studies included periacetabular osteotomy and surgical hip dislocation in which peripheral nerve blocks were associated with an 18% reduction in pain on postoperative Day 2, a 48% reduction in cumulative opioid use on postoperative Day 2 and a 40% reduction in hospital stay. Due to the low sample size of the periacetabular osteotomy and surgical hip dislocation studies, we were unable to determine the significant difference between the means. Due to significant between-study heterogeneity, additional studies with congruent outcome measures need to be conducted to determine the efficacy of regional anesthesia in hip arthroscopy, periacetabular osteotomy and surgical hip dislocation.

## INTRODUCTION

Hip preservation surgeries have grown in popularity over the past decade in treating various pre-arthritic hip conditions in adolescents and adults [1, 2]. Hip preservation procedures include hip arthroscopy, periacetabular osteotomy (PAO), proximal femoral osteotomy, cartilage restoration and surgical hip dislocation (SHD) [2, 3]. These procedures aim to improve mechanical function in certain pathologies, including femoroacetabular impingement, labral tears, chondral lesions of the acetabulum and acetabular dysplasia [1, 4, 5]. With new developments in hip preservation surgery, anesthetic techniques must keep pace with surgical advancements.

Regional anesthesia, which includes peripheral nerve blocks (PNBs), local infiltrative anesthesia (LIA) and combined spinal epidural anesthesia, can be helpful in managing moderate-to-severe postoperative pain in patients undergoing hip preservation surgery [6–8]. Adjunct medications such as non-steroidal anti-inflammatory drugs (NSAIDs) and opioid-based medications are also common pain interventions used to supplement general anesthesia (GA) in hip preservation surgeries. This multimodal approach to pain relief has reduced pain and postanesthesia care unit (PACU) length of stay (LOS) in hip fracture and hip arthroscopy studies [8–11]. Previous studies have also suggested that a multimodal pain regimen can reduce postoperative opioid use, which has become increasingly important in the current opioid crisis [8, 12–14]. However, there is limited literature directly comparing the efficacy of PNB, LIA and adjunct medication along with GA in hip preservation surgery [8–11].

This study aims to provide an updated comprehensive systematic review of perioperative pain management in hip arthroscopy as well as a new perspective on anesthetic regimens in PAO and SHD. Part I of this systematic review investigates perioperative pain management in hip arthroscopy. Part II investigates perioperative pain management in PAO and SHD.

## METHODS

### Inclusion criteria

#### Types of studies

The literature search performed in this review was limited to original published reports concerning perioperative anesthesia in patients undergoing hip arthroscopy, PAO and SHD. The data analysis did not include abstracts from scientific meetings, unpublished reports, case reports and review articles.

#### Types of participants

Male and female humans of all ages undergoing hip arthroscopy, PAO or SHD were included in this study.

#### Types of intervention

Interventions included regional anesthesia, adjunct medication and GA to treat perioperative pain in patients undergoing hip arthroscopy, PAO and SHD.

#### Types of outcome measures

Pain measurements, reported as visual analog scale (VAS), numerical rating scale (NRS) or Defense and Veterans Pain Rating Scale (DVPRS), were primary outcomes for both the hip arthroscopy and PAO/SHD sections. Opioid consumption [morphine milligram equivalent (MME)] and PACU LOS were secondary outcomes for hip arthroscopy and PAO/SHD. Complications of each block were included.

#### Quality assessment of included studies

The quality and risk of bias of randomized controlled trials (RCTs) were assessed using the Jadad scale [15]. The quality and risk of bias of retrospective review articles were assessed using the methodological index for non-randomized studies (MINORS) criteria [16].

### Search strategy

A systematic review was conducted according to the Preferred Reporting Items for Systematic Reviews and Meta-Analysis guidelines [17]. Two reviewers (E.M.B. and J.A.A.) independently conducted a literature search throughout the PubMed, MEDLINE (Ovid) and Embase databases between January 2022 and December 2022 with final inclusion determined by the senior authors (W.A. and M.M.H.). The date range used to retrieve studies included January 2000 to December 2022. The search for anesthetic techniques in hip arthroscopy included the following terms: block, peripheral nerve block, anesthesia, anesthetic, analgesia, hip, hip joint, PENG and arthroscopy. Search for anesthetic techniques in PAO and SHD included the following terms: surgical hip dislocation, periacetabular osteotomy, PAO, epidural block, quadratus lumborum block, lumbar plexus block, anaesthesia, anesthetic, anesthesia and nerve block. A more detailed description of the search strategy is included in the Appendix.

### Statistical analysis

Due to significant heterogeneity between studies, studies were manually grouped by similar outcome measures. Two-sample *t*-tests and analysis of variances (ANOVA) were used to calculate the significant difference between means, which was set at alpha = 0.05. Due to the low sample size of PAO/SHD anesthetic studies, the *P*-value was not calculated in this section.

## PART I: ARTHROSCOPY

### Part I: results

**Fig. 1.**
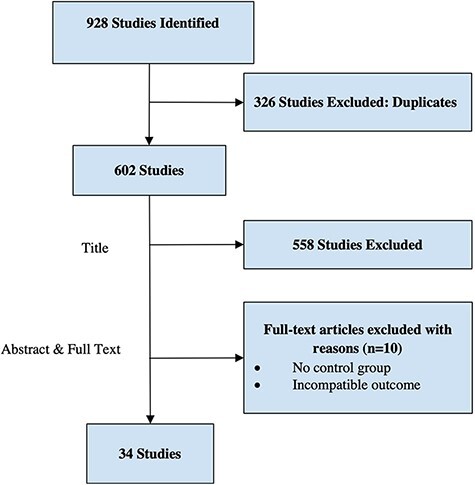
The Preferred Reporting Items for Systematic Reviews and Meta-Analysis (PRISMA) flow chart.

This comprehensive literature search for articles related to pain management in arthroscopic hip preservation surgeries initially identified 928 papers, of which 44 were included for abstract and full-text analysis. After exclusions, 34 studies comprised the final analysis (Fig. 1). The details of these studies are outlined in Table I.

**Table I.** Summary of perioperative pain management studies in hip arthroscopy

									*Outcome*				
*Study*	*Study type*	*Level of evidence*	*Peripheral nerve block*	*Peripheral nerve block timing*	*Local infiltrative anesthesia or adjunct medication*	*Number of patients*	*Age (mean years)*	*Sex*	*Mean pain score at discharge (NRS/VAS/DVPRS)*	*Opioid use in PACU (MME)*	*PACU LOS (min)*	*Complications*	*MINORS/Jadad*
Wolff *et al.* [33]	Retrospective review	3	No block	NR	NR	55	34.51	18 M 37 F	3.55 (NRS)	7.02	108.6	None	17.5/24
			FICB (40 ml 0.375% ropivacaine and 4 mg dexamethasone)	Preoperative	NR	30	30.57	11 M 19 F	4.08 (NRS)	8.8	112.2	None	
			LPB (40 ml 0.375% ropivacaine and 4 mg dexamethasone)	Preoperative	NR	60	35.45	19 M 41 F	2.38 (NRS)	7.16	111.5	Seizure (*n* = 1)	
									***P* < 0.001**	*P* = 0.531	*P* = 0.928		
Xing *et al.* [18]	RCT	1	No block	NR	NR	23	31	14 M 9 F	NR	NR	252.5	None	5/5
			FNB (20 ml 0.5% ropivacaine and 2.5 μg ml^−1^ epinephrine)	Preoperative	NR	27	32	21 M 6 F	NR	NR	246	Falls (*n* = 6)	
Philippi *et al.* [14]	Retrospective review	3	No block	NR	NR	22	33.64	18 M 32 F	NR	14.08	NR	NR	18/24
			FNB	Postoperative	NR	28							
			No block	NR	LIA 20 ml of 0.25% bupivacaine–epinephrine (1:200 000)	33	37.44	14 M 36 F	NR	14.51	NR	NR	
			FNB	Postoperative	LIA 20 ml of 0.25% bupivacaine–epinephrine (1:200 000)	17							
Blackwell *et al.* [43]	Retrospective series	3	QLB	Preoperative	15 mg of Toradol and ropivacaine 0.5%	43	34.0	NR	3.27 (DVPRS)	20.7	116	None	17/24
			FNB/FICB	Preoperative	15 mg of Toradol and ropivacaine 0.5%	58	37.6	NR	4.98 (DVPRS)	28.7	148	Fall (*n* = 2)	
									***P* < 0.001**	***P* = 0.03**	***P* < 0.001**		
Scanaliato *et al.* [19]	RCT	1	LPB	Preoperative	NR	32	38.6	12 M 20 F	NR	11.11	124.39	NR	4/5
			PENG	NR	NR	32	37.9	15 M 17 F	NR	8.72	137.37	NR	
										P = 0.39	P = 0.43		
Yuan *et al.* [44]	RCT	1	QLB (0.4% ropivacaine 0.4 ml/kg)	Preoperative	NR	40	36.7	19 M 21 F	4 (VAS)	NR	NR	NR	5/5
			No block	NR	NR	40	36.5	20 M 20 F	7 (VAS)	NR	NR	NR	
									***P* < 0.001**				
Childs *et al.* [34]	Retrospective review	3	FNB (0.5% bupivacaine, 1:200 000 epinephrine)	Preoperative	NR	105	33.4	38 M 76 F	3.55 (VAS)	10.43^a^	NR	Peripheral neuritis (*n* = 26) Falls (*n* = 19)	17/24
			No block	NR	IA (300 mg ropivacaine (0.5%) with epinephrine, 30 mg ketorolac, 5 mg morphine)	88	31.3	27 M 61 F	4.28 (VAS)	12.53^a^	NR	Peripheral neuritis (*n* = 2) Falls (*n* = 5)	
									***P* = 0.03**	*P* = 0.11	NR		
Glomset *et al.* [20]	RCT	2	FICB (60 ml 0.35% ropivacaine at a dose of 3 mg/kg, with adjuvants of 100 μg clonidine (per 60 ml) and epinephrine 1:400 000)	Preoperative	NR	41	40.6	15 M 26 F	4.4 (VAS)	12.7	150.0	NR	3/5
			No block	NR	LIA (20 ml of 0.5% ropivacaine)	43	36.8	13 M 30 F	4.9 (VAS)	14.8	151.6	NR	
									*P* = 0.51	*P* = 0.29	*P* = 0.92		
Turner *et al.* [35]	Retrospective review	3	Neuraxial anesthesia (0.5% bupivacaine)	Preoperative	IA 20-cc 0.25% bupivacaine	77	29.3	34 M 43 F	3 (NRS)	18.2	258	Numbness or (weakness) = 18 Spinal headache = 4 Any anesthetic complications = 5	18/24
			No block	NR	IA 20-cc 0.25% bupivacaine	52	26.3	20 M 32 F	4 (NRS)	31.2	264	Any complications (*n* = 12)	
									***P* = 0.013**	***P* < 0.001**			
Philippi *et al*. [21]	RCT	1	No block	NR	LIA (20 ml of 0.25% bupivacaine–epinephrine)	36	36.3	11 M 25 F	3.36 (NRS)	11.91	124.5	NR	4/5
			No block	NR	NR	37	33.3	10 M 27 F	3.86 (NRS)	15.11	136.8	NR	
									*P* = 0.18	*P* = 0.19	*P* = 0.09		
Huang *et al.* [22]	RCT	1	FICB (35–40 ml of ropivacaine 0.35%)	Preoperative	NR	27	42.4	12 M 15 F	3.0 (VAS)	31	NR	NR	3/5
			No block	NR	NR	33	41.2	7 M 26 F	3.3 (VAS)	23.9	NR	NR	
									*P* = 0.651	*P* = 0.958			
McCrum *et al.* [45]	Retrospective matched cohort	3	QLB (20–30 ml 0.5% ropivacaine, dexmedetomidine 20–30 µg and dexamethasone 4 mg)	Preoperative	NR	28	37	11 M 17 F	2.57 (VAS)	6.53	NR	Numbness (*n* = 1)	17/24
			No block	NR	NR	28	36	8 M 20 F	4.18 (VAS)	14.02	NR	None	
									***P* = 0.015**	***P* < 0.001**			
Haskins *et al.* [23]	RCT	1	QLB (30 ml 0.5% bupivacaine and 2 mg dexamethasone)	Preoperative	NR	48	36	24 M 24 F	NR	NR	276	NR	17/24
			No block	NR	NR	48	36	22 M 26 F	NR	NR	252	NR	
											*P* = 0.098		
Behrends *et al.* [24]	RCT	1	FICB (40 ml ropivacaine 0.2%)	Preoperative	Intracapsular (10 ml of 0.2% ropivacaine)	38	35	23 M 15 F	3 (NRS)	15	123	Falls (*n* = 4)	5/5
			No block	NR	Intracapsular (10 ml of 0.2% ropivacaine)	37	32	17 M 23 F	3 (NRS)	16	128	Falls (*n* = 1)	
Badiola *et al.* [25]	RCT	1	FICB (30ml 0.25% bupivacaine with 1:200 000 epinephrine)	Postoperative	None	25	39.8	9 M 16 F	NR	20.8	139.72	None	4/5
			LPB (30ml 0.25% bupivacaine with 1:200 000 epinephrine)	Postoperative	None	23	38.8	7 M 16 F	NR	16.98	165.04	Weakness (*n* = 1)	
										***P* = 0.02**	*P* = 0.419		
Wilson *et al.* [26]	RCT	1	QLB (40 ml ropivacaine 0.25%)	Preoperative	NR	22	29.8	8 M 14 F	5.79 (VAS)	8.1	99.9	NR	5/5
			No block	NR	NR	24	37.1	10 M 14 F	5.92 (VAS)	11.3	110.3	NR	
									*P* = 0.84	***P* = 0.038**	*P* = 0.26		
Alrayashi *et al.* [36]	Retrospective review	3	FICB (ropivacaine 0.2%)	Preoperative	NR	275	21.3	97 M 178 F	NR	NR	93	NR	16/24
			No block	NR	NR	441	21.4	133 M 308 F	NR	NR	108	NR	
											***P* < 0.001**		
Ward *et al.* [27]	RCT	2	FNB (25 ml 0.25% bupivacaine with 1:200 000 epinephrine)	Postoperative	NR	20	35.8	10 M 10 F	NR	NR	177.85	NR	2.5/5
			No block	NR	NR	20	41.8	5 M 11 F	NR	NR	216	NR	
											***P* < 0.001**		
Cogan *et al.* [37]	Retrospective review	3	No block	NR	IA (10 mg morphine and 100 mg clonidine)	22	38	7 M 14 F	NR	23	134	None	18/24
			No block	NR	NR	21	34	6 M 16 F	NR	40	133	None	
										***P* = 0.02**	*P* = 0.56		
Purcell *et al.* [28]	RCT	1	FICB (40 cc 0.25% plain bupivacaine)	Preoperative	NR	33	32.8	12 M 21 F	4 (DVPRS)	NR	NR	Falls (*n* = 3)	5/5
			FICB (266 mg liposomal bupivacaine and 20 cc 0.5% plain bupivacaine)	Preoperative	NR	37	30.1	25 M 12 F	4 (DVPRS)	NR	NR	Falls (*n* = 1)	
									*P* = 0.61		*P* = 0.43	*P* = 0.33	
Purcell *et al.* [38]	Retrospective review	3	FICB (266 mg liposomal bupivacaine and 20 cc 0.5% plain bupivacaine)	Preoperative	NR	34	34.7	23 M 11 F	2.41 (DVPRS)	32.53	NR	None	17/24
			FICB (40 cc 0.25% plain bupivacaine)	Preoperative	NR	34	32.4	15 M 19 F	2.88 (DVPRS)	29.46	NR	None	
YaDeau *et al.* [29]	RCT	1	LPB (30 ml 0.25% bupivacaine, 1:200 000 epinephrine)	Preoperative	NR	41	33	20 M 21 F	3.3 (NRS)	21	NR	Falls (*n* = 2) Epidural spread and urinary retention (*n* = 1)	5/5
			CSE (intrathecal mepivacaine, 45–60 mg (1.5%)	NR	NR	41	37	21 M 20 F	4.2 (NRS)	29	NR	Oxygen desaturation (*n* = 1)	
									***P* = 0.048**	*P* = 0.051			
Kahlenberg *et al.* [30]	RCT	1	No block	NR	Celecoxib (400 mg)	50	34.2	24 M 26 F	NR	15.33	152.96	NR	5/5
			No block	NR	NR	48	35.8	19 M 29 F	NR	15.42	172.96	NR	
										*P* = 0.48	***P* = 0.04**		
Kinjo *et al.* [39]	Retrospective review	3	QLB	Preoperative	IA (10 ml of 0.2% ropivacaine)	15	35	5 M 10 F	NR	NR	114	NR	18/24
			No block	NR	IA (10 ml of 0.2% ropivacaine)	54	35	30 M 25 F	NR	NR	120	NR	
Zhang *et al.* [31]	RCT	1	No block	NR	Celecoxib (200 mg)	27	41	11 M 13 F	7.23 (VAS)	NR	147	NR	5/5
			No block	NR	NR	26	43.5	14 M 15 F	7.46 (VAS)	NR	152	NR	
Shlaifer *et al.* [32]	RCT	1	No block	NR	PA bupivacaine (20 ml 0.5%): preoperative IA bupivacaine (20 ml 0.5%): postoperative	21	39.6	14 M 7 F	NR	5	123	NR	5/5
			No block	NR	IA bupivacaine (20 ml 0.5%): preoperative	21	36	11 M 10 F	NR	6.5	213	NR	
					IA bupivacaine (20 ml 0.5%): postoperative					*P* = 0.558	***P* = 0.007**		
Kazum *et al.* [46]	Prospective case series	3	No block	NR	PA bupivacaine (20 ml 0.5%): preoperative IA bupivacaine (20 ml 0.5%): postoperative	20	38.55	16 M 6 F	NR	5.75	NR	NR	22/24
			No block	NR	PA bupivacaine (20 ml 0.5%): preoperative	32	33.47	21 M 11 F	NR	7.73	NR	NR	
										*P* = 0.56			
Dold *et al.* [40]	Retrospective review	3	FNB (15–25 ml ropivacaine 0.33–0.75%)	Preoperative	NR	56	33.07	37 M 19 F	NR	2.04	85.96	NR	18/24
			No block	NR	NR	40	34.18	22 M 18 F	NR	4	81.53	NR	
										*P* = 0.25	*P* = 0.44		
Potter *et al.* [47]	Prospective case series	4	FICB (30 ml 0.25% bupivacaine, 5 μg/ml epinephrine)	Postoperative	NR	53	37.1	15 M 38 F	2.8 (VAS)	4.4	102	Admission for pain (*n* = 3)	22/24
			No block	NR	NR	54	34.9	26 M 28 F	3.4 (VAS)	4.4	97	NR	
									*P* = 0.15	*P* = 0.98	*P* = 0.08		
Kolaczko *et al.* [41]	Retrospective review	3	QLB [30 cc 0.5% bupivacaine or ropivacaine with epinephrine (1 in 200 000) and dexamethasone 4 mg]	Preoperative	NR	241	30	96 M 175 F	4 (VAS)	13.3	176.3	Numbness (*n* = 3) Dizziness and blurriness (*n* = 1)	18/24
			Multimodal	NR	NR	196	27.8	77 M 136 F	4 (VAS)	12.4	137.4	Numbness (*n* = 1)	
									*P* = 0.83	*P* = 0.29	***P* < 0.001**		
Schroeder *et al.* [42]	Retrospective review	3	LPB (20–30 ml of 0.5% ropivacaine with 3 µg/ml epinephrine)	Preoperative	NR	118	41.1	39 M 79 F	NR	NR	240	NR	18/24
			No block	NR	NR	118	38.7	47 M 71 F	NR	NR	217.5	NR	
											***P* = 0.044**		
Amato *et al.* [48]	RCT	1	PENG (20 ml of 0.5% ropivacaine)	NR	NR	34	32.5	15 M 19 F	NR	7.5	NR	NR	5/5
			No block	NR	NR	34	29.4	14 M 20 F	NR	8.7	NR	NR	
Kollmorgen *et al.* [49]	Retrospective review	3	PENG	NR	NR	25	26.5	12 M 13 F	4.5	NR	81.5	NR	18/24
			No block	NR	NR	25	25.52	11 M 14 F	5.4	NR	95.8	NR	
Yusupov *et al.* [50]	Retrospective review	3	PENG (15–20 ml of bupivacaine or ropivacaine)	NR	NR	28	36	10 M 18 F	NR	14.4	129	NR	17/24
			No block	NR	NR	25	31	10 M 15 F	NR	31.2	161	NR	

CSE: combined spinal epidural; PA: periacetabular; IA: intraarticular; NR: not reported. Bold values denote statistical significance at the *P* < 0.05 level.

^a^Converted from mg oxycodone to MME.

Of the 34 studies analyzed, 17 were RCTs [18–32, 44], 13 were retrospective reviews [14, 33–42], 1 was a retrospective series [43] and 3 were case series (one retrospective and two prospective) [45–47]. Fifteen (44.1%) studies were considered Level 1 evidence, 2 (6.5%) were Level 2, 16 (47.1%) were Level 3 and 1 (3.2%) was Level 4 evidence. The quality of all RCTs was assessed using the Jadad criteria, while retrospective reviews were assessed with MINORS criteria. The mean MINORS score was 17.5 ± 0.63, and the mean Jadad score was 4.2 ± 0.94.

The 34 studies included a total of 3657 patients undergoing hip arthroscopy. The mean patient age at the time of surgery was 33.30 ± 7.6 years. Females comprised 60.4% (*n* = 2208 of 3657) of patients. One study did not report patient sex [43]. In all studies, the mean PACU VAS at the time of discharge was 4.6. The mean PACU NRS at the time of discharge was 3.3. The mean PACU DVPRS was 3.3. The mean PACU opioid consumption was 13.2 MME. The mean PACU LOS was 154 min. The mean pain scores, opioid use and LOS for each block type are outlined in Table II.

**Table II.** Hip arthroscopy outcomes by intervention

	*Mean pain scores*					
*Intervention*	*VAS*	*DVPRS*	*NRS*	*Mean opioid use in PACU (MME)*	*PACU LOS (min)*	*Complication rate*
No block	5.08	NR	3.89	17.18	158.61	Numbness: 0.8% Oxygen desaturation: 0.8%
All PNB	3.84	NR	3.25	13.0	150.99	NR
QLB	4.09	3.27	NR	9.31	167.05	Numbness: 1.05% Dizziness: 0.26% Constipation: 0.26%
FNB	3.55	NR	NR	6.24	169.94	Falls: 11.70% Peripheral neuritis: 9.81%
LPB	NR	NR	2.84	14.06	160.23	Seizure: 0.36% Weakness: 0.36% Epidural spread and urinary retention: 0.36% Falls: 0.73%
FICB	3.40	3.44	4.08	15.54	119.38	Falls: 0.65% Admission for abdominal pain: 0.49%
PENG	4.5	NR	NR	10.95	105.25	NR
LIA	4.59	NR	3.18	10.91	137.63	Falls: 1.22% Peripheral neuritis: 0.41% Numbness: 0.20% Oxygen desaturation: 0.20%
Preoperative celecoxib	5.52	NR	NR	15.33	149.98	NR

NR: not reported.

All patients undergoing hip arthroscopy received GA; 65.1% of patients (*n*= 2381 of 3657) received some form of regional or adjunct anesthesia in addition to GA, while 34.89% of patients (*n* = 1276 of 3657) received GA alone. Regional interventions included fascia iliaca compartment block (FICB) (17.1%, *n* = 618) [20, 22, 25, 26, 33, 47], lumbar plexus block (LPB) (7.6%, *n* = 274) [19, 25, 29, 33, 42], femoral nerve block (FNB) (7.3%, *n* = 265) [18, 27, 34, 40], quadratus lumborum block (QLB) (11.7%, *n* = 422) [23, 26, 41, 43–45], pericapsular nerve group (PENG) block (3.3%, *n* = 119) and LIA (12.6%, *n* = 459) [14, 19–21, 24, 32, 34, 37, 39, 46, 48–50]. Combination blockade was performed in 4.07% of patients (*n* = 147). Combinations included QLB + LIA (0.42%, *n*= 15) [39], FNB + LIA (0.48%, *n*= 17) [35], FICB + LIA (1.1%, *n* = 38) [24] and neuraxial + LIA (2.1%, *n* = 77) [35]. Adjunct medication included celecoxib (2.1%, *n* = 77) [30, 31].

**Fig. 2.**
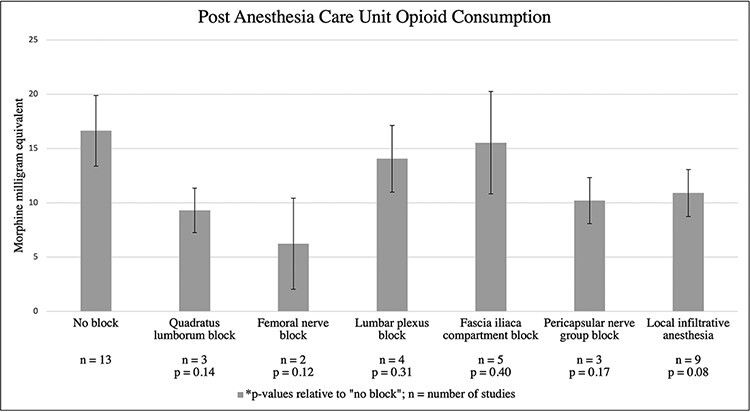
The opioid use by block type.

PNBs were associated with a significant reduction in pain score (*P* = 0.037) compared with GA alone. However, no pain control modality was associated with a significant difference in opioid use (*P* = 0.127) or LOS (*P* = 0.251) compared with GA alone, as depicted in Fig. 2. A comparison of each block category with a level of significance is analyzed in Table III.

**Table III.** Two-sample t-test and analysis of variances based on pain control modality

	P*-value*			
	*Pain scores*			
*Intervention*	*VAS*	*NRS*	*Opioid use (MME)*	*PACU LOS (min)*
PNB versus GA alone	***P* = 0.037**	*P* = 0.177	*P* = 0.127	*P* = 0.251
PNB versus LIA	*P* = 0.173	*P* = 0.459	*P* = 0.233	*P* = 0.369
LIA versus GA alone	*P* = 0.346	*P* = 0.053	*P* = 0.148	*P* = 0.210
FNB versus FICB versus QLB versus LPB versus PENG	NR	NR	*P* = 0.593	*P* = 0.530

NR: not reported. Bold values denote statistical significance at the *P* < 0.05 level.

A total of 111 complications were reported in 12 studies, giving an overall complication rate of 3.10% [18, 24, 25, 28, 29, 33–35, 41, 43, 45, 47]. The reported complications included falls (36.6%, *n*= 41) [18, 24, 28, 29, 34, 43], peripheral neuritis (25.0%, *n* = 28) [34], weakness (17.0%, *n* = 19) [25, 35, 45], numbness (5.4%, *n* = 6) [25, 41, 45], any anesthetic (4.5%, *n* = 5) [35], spinal headache (3.6%, *n* = 4) [35], oxygen desaturation (2.68%, *n* = 3) [29], admission for pain (2.68%, *n* = 3) [47], urinary retention due to epidural spread (0.89%, *n* = 1) [29] and seizure (0.89%, *n* = 1) [33].

### Part I: discussion

The present review analyzes the various pain management strategies in hip preservation surgeries, distinguishing between those used in hip arthroscopy and those used in open PAO or SHD. In hip arthroscopy, there was a significant difference in VAS pain score at discharge with PNB compared with GA alone, but there was no significant difference in opioid usage or LOS among patients receiving FNB, FICB, LPB, QLB, GA, neuraxial anesthesia, NSAIDs or LIA. No single technique provided superior relief compared with other blocks or GA alone. Falls and peripheral neuritis were the most common complications and were most frequently associated with FNB in hip arthroscopy.

PNBs have increased in popularity along with advances in hip preservation surgery. In light of the current opioid epidemic, reducing postoperative opioid use remains an essential goal for enhancing surgical recovery and preventing future abuse. A prior study found that increased opioid dosing in the early postoperative period following total joint arthroplasty was associated with an increased risk for abuse [51]. Several individual studies in this review reported superior pain control and reduction in opioid use with regional anesthesia compared with GA alone [19, 29, 34, 35, 43–45, 48–50]. When analyzing the outcome measures of all included studies, our manually grouped data suggest that PNB with GA significantly reduces VAS pain scores compared with GA alone (*P* = 0.037). However, no other type of anesthesia was associated with a significant reduction in pain or opioid use. Despite many studies showing a reduction in opioid use, the sample size of the available studies is rather small with marked between-study heterogeneity and does not reach a level of significance. Additional RCTs with congruent outcome measures would allow a more robust meta-analysis that would elucidate the true effect of regional anesthesia on pain and opioid use.

Reducing pain and postoperative complications has been associated with a shorter LOS, which is important for reducing hospital costs [52, 53]. Several studies in this review found a significant reduction in LOS in patients receiving a regional block in hip arthroscopy [27, 30, 32, 36, 41–43], which highlights the potential for the regional blockade to reduce hospital costs by decreasing patient time in the PACU. However, when including the outcomes of all studies, most found that regional anesthesia did not reduce the overall LOS in patients undergoing hip arthroscopy. While this may be due to the lack of additional benefit from PNB in reducing LOS, there was also significant between-study heterogeneity in LOS as an outcome measure, making the true effect of PNB on LOS difficult to analyze. This highlights the need for future prospective studies with large sample sizes, perhaps multicenter prospective studies, to truly elucidate the effect regional anesthesia has on pain, opioid use and LOS.

The overall complication rate in patients undergoing hip arthroscopy was low (3.10%), with falls accounting for 37% of complications in our study. Of the 31 falls reported, FNB was responsible for 25 (80.65%). This increased rate of falls with FNB in hip arthroscopy is consistent with the existing literature highlighting this risk [18, 24, 29, 34]. In addition to falls, peripheral neuritis accounted for 25% of the reported complications in this study. Previous studies estimate that ∼0.4–13.3% of patients experience neurological complications when undergoing hip arthroscopy with a PNB [54], with long-term nerve injury occurring <1% of the time [55]. While not an independent risk factor, the intrafascicular, high-pressure injections associated with PNB have been linked to peripheral nerve injury [54]. Ultrasound guidance continues to be the safest way to perform PNB to avoid neurological complications [56].

In addition to providing safe guidance for performing PNB, ultrasound guidance has recently been used to perform targeted anesthesia techniques like LIA [56, 57]. Given the complex innervation of the hip joint, a complete sensory blockade is difficult to achieve. The effective blockade of the hip joint must include the femoral nerve, sciatic nerve, superior gluteal nerve and obturator nerve, each of which innervates a unique component of the hip [58]. LIA can be beneficial in providing targeted pain relief in areas that are not anesthetized by PNB. In our review, 17% of patients underwent LIA alone or combined with a PNB. Despite some studies showing improvement in pain control, opioid use and LOS compared with GA [32, 34, 35], most studies suggest that LIA alone or in combination with PNB did not outperform other regional anesthesia or GA alone in pain control, opioid use or LOS in hip arthroscopy procedures.

Moreover, a recent technique developed by Philip Peng may provide superior relief by anesthetizing smaller distal sensory nerve branches with less risk of motor blockade [59]. When compared with FNB, the PENG approach provided superior pain control and preserved quadriceps strength for patients undergoing hip fracture surgery [60]. Three studies included in this review demonstrate a significant reduction in pain, opioid use or LOS with the PENG approach compared with GA alone [48–50]. Future prospective studies need to be conducted in order to perform a more robust meta-analysis on the efficacy of the PENG technique in hip arthroscopy.

This study is not without limitations. Due to the significant heterogeneity of outcome measures between studies, we could not pool the data for meta-analysis. The authors instead pooled similar outcome measures to standardize the data before performing a two-sample *t*-test and ANOVA to determine a significant difference between the means. Another limitation is that various comorbidities among patients may not have been included in individual studies. Previous studies have demonstrated that various comorbidities such as disability, opioid tolerance, surgical complexity, intraoperative fluid administration and perioperative complications affect outcome measures such as pain, opioid use and LOS [61]. One study also suggests that higher levels of preoperative psychological distress may increase PACU opioid requirements [47], an outcome measure not included in this review. The inability to control for these comorbidities could have added confounding factors to our data.

### Part I: conclusion

In this study, PNBs were associated with a significant decrease in VAS pain scores at discharge compared with GA alone, but there was no significant difference in PACU opioid use or LOS among perioperative pain regimens in hip arthroscopy patients. While the efficacy of regional anesthesia in hip preservation surgery has not been comprehensively shown in the literature, it continues to be utilized in many centers. Most studies in this review assess the efficacy of regional anesthesia in the acute (<24 h) phase of surgical recovery. However, differences in efficacy need to be studied in a more robust fashion, preferably looking at both long- and short-term functional outcomes, which current literature is lacking. It is the hope of the authors of this review that future multicenter prospective studies will be conducted to explore these remaining questions.

## PART II: PAO/SHD

### Part II: results

**Fig. 3.**
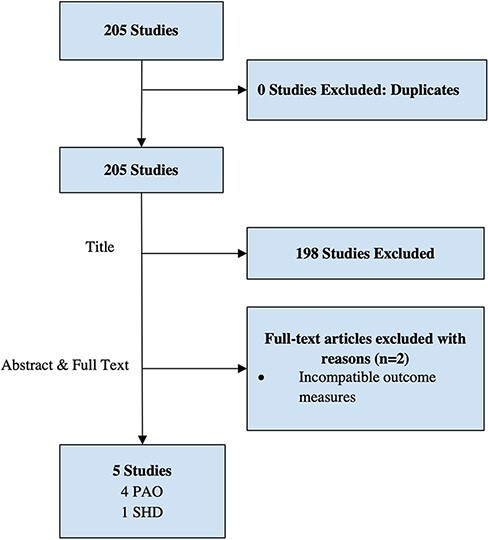
The Preferred Reporting Items for Systematic Reviews and Meta-Analysis (PRISMA) flow chart.

We initially found 205 studies. Seven were included for abstract and full-text analysis. Two were excluded from the final analysis. Of the five studies included, four analyzed anesthesia in PAO, and one analyzed anesthesia in SHD (Fig. 3).

This review investigated a total of three RCTs, one retrospective reviews and one ambidirectional cohort. Studies accessed at Level 1 evidence made up 40% (*n* = 2) and Level 3 made up 40% (*n* = 2). One study did not report the level of evidence. The mean MINORS score was 18.50 ± 4.94, and the mean Jadad score was 4.33 ± 0.58. Details on included studies are outlined in Table IV.

**Table IV.** Summary of perioperative pain management studies in periacetabular osteotomy and surgical hip dislocation

									*Outcome*			
*Study*	*Study type*	*Level of evidence*	*Peripheral nerve block*	*Peripheral block timing*	*LIA or adjunct medication*	*Number of patients*	*Age (mean years)*	*Sex*	*Postoperative pain scores (NRS/VAS)*	*Cumulative postoperative opioid consumption okay (MME)*	*Time until discharge (days)*	*MINORS/JADAD*
Albertz *et al.* [63]	Ambidirectional cohort	3	FICB (0.2% ropivacaine ml/kg up to 40 ml)	Preoperative	NR	16	17^a^	1 M 15 F	POD0: 4.47 (NRS) POD1: 3.91 (NRS) POD2: 3.54 (NRS)	NR	2.88	22/24
			Lumbar epidural block (0.2% ropivacaine infusion until POD2)	Preoperative	NR	16	17^a^	1 M 15 F	POD0: 4.11 (NRS) POD1: 4.67 (NRS) POD2: 4.47 (NRS)	NR	4.38	
											***P* < 0.001**	
Löchel *et al.* [62]	RCT	1	TAP (20 ml 0.75% ropivacaine)	Preoperative	NR	21	NR	NR	POD0: 3.7 (NRS) POD1: 2.9 (NRS) POD2: 4.5 (NRS)	POD0: 2.49 POD1: 15.27 POD2: 24.15	8.7	5/5
			No block	NR	NR	20	NR	NR	POD0: 4.6 (NRS) POD1: 4.2 (NRS) POD2: 5.1 (NRS)	POD0: 8.96 POD1: 25.56 POD2: 45.4	8.8	
Bech *et al.* [65]	RCT	1	No block	NR	LIA (75 ml ropivacaine)	26	35	9 M 17 F	POD0: 2.9 (VAS) POD1: 3.1 (VAS) POD2: 2.4 (VAS)	NR	NR	4/5
			No block	NR	NR	27	31	3 M 24 F	POD0: 3.8 (VAS) POD1: 3.5 (VAS) POD2: 3.1 (VAS)	NR	NR	
Steinthorsdottir *et al.* [66]	RCT	NR	No block	NR	8 mg dexamethasone	32	29	8 M 24 F	POD0: 3.9 (NRS) POD1: 4.1 (NRS) POD2: 3.7 (NRS)	NR	3.2	4/5
			No block	NR	48 mg dexamethasone	32	28	5 M 27 F	POD0: 2.8 (NRS) POD1: 3.6 (NRS) POD2: 3.2 (NRS)	NR	3.25	
Novais *et al.* [64]	Retrospective review	3	NR	NR	LIA (ropivacaine 0.2% 0.3 ml/kg, morphine 0.1 mg/kg, methylprednisolone 0.5 mg/kg and saline with epinephrine)	20	16.8	12 M 8 F	NR	NR	1.58	15/24
			Lumbar epidural (ropivacaine 0.075–0.2% or bupivacaine 0.075–0.1%)	Preoperative	NR	72	16.3	20 M 52 F	NR	NR	3	
			No block	NR	Patient-controlled anesthetic	29	17.4	8 M 21 F	NR	NR	2.54	

NR: not reported. Bold values denote statistical significance at the *P* < 0.05 level.

a Reported as median.

The five included studies had a total of 311 patients undergoing PAO or SHD. The mean patient age at the time of surgery for PAO was 30.78 ± 3.18 and 16.83  ± 0.55 years for SHD. Females comprised 75.19% (*n* = 203 of 270) of patients. One study did not report patient sex [62]. The mean NRS on postoperative Day 2 (POD2) was 3.19. The mean cumulative opioid consumption following surgery was 12.93 MME. The mean LOS was 4.26 days.

Overall, 54.98% of patients received regional anesthesia (*n* = 171 of 311). Regional interventions included FICB (5.14%, *n* = 16) [63], lumbar epidural anesthesia, (28.30%, *n* = 88) [63, 64], transversus abdominis plane (TAP) (6.75%, *n* = 21) [62] and LIA (14.79%, *n* = 46) [64, 65]. Adjunct medication included dexamethasone (20.58%, *n* = 64) [66]. A total of 11 complications occurred in 311 patients, giving an overall complication rate of 3.54% [64].

Löchel *et al*. found a significant difference in pain control with TAP on POD1, but there was no significant difference in pain control in all other studies or time points [62]. Two studies reported a significant decrease in opioid consumption [62, 66]. Löchel *et al*. found that the mean opioid consumption was significantly lower in the TAP group compared with GA alone. Steinthorsdottir *et al*. found a significant decrease in opioid consumption using 48 mg dexamethasone compared with 8 mg dexamethasone during the first 4 PODs [66]. Two studies reported significantly shorter lengths of hospital stay [63, 64]. FICB was associated with a significantly reduced LOS compared with FNB, and LIA was associated with a significantly reduced length of hospital stay compared with lumbar epidural block and PCA [63, 64]. There were no specific complications reported. These data are outlined in Table V.

**Table V.** Periacetabular osteotomy/surgical hip dislocation outcomes by intervention

	*Mean pain scores*			
*Intervention*	*VAS*	*NRS*	*Cumulative opioid consumption (MME)*	*Mean time until discharge (days)*
No block	POD0: 3.8 POD1: 3.5 POD2: 3.1	POD0: 4.6 POD1: 5.2 POD2: 4.1	POD0: 8.96 POD1: 25.56 POD2: 45.40	8.8
FICB	NR	POD0: 4.47 POD1: 3.91 POD2: 3.54	NR	2.88
Lumbar epidural	NR	POD0: 4.11 POD1: 4.67 POD2: 4.47	NR	4.38
TAP	NR	POD0: 3.70 POD1: 2.90 POD2: 4.50	POD0: 2.49 POD1: 15.27 POD2: 24.15	8.7
LIA	NR	NR	NR	2.37
8 mg dexamethasone	NR	POD0: 3.9 POD1: 4.1 POD2: 3.7	NR	3.2
48 mg dexamethasone	NR	POD0: 2.8 POD1: 3.6 POD2: 3.2	NR	3.25

NR: not reported.

### Part II: discussion/conclusion

To our knowledge, there have been no previous systematic reviews assessing the efficacy of regional anesthesia in hip PAO or SHD. While we were unable to determine the level of significance with the currently limited literature, our preliminary findings encourage the continued exploration of the efficacy of regional blockade in PAO and SHD. With the inclusion of this section, it is the hope of these authors that additional prospective studies with congruent outcome measures be conducted to evaluate for a significant improvement in pain, opioid use and LOS with regional anesthesia in PAO and SHD.

## Data Availability

All data were derived from public domain resources.

## APPENDIX

### Part I: Arthroscopy Search Strategy for all Databases:

(block OR peripheral nerve block) OR (PENG) OR (Pericapsular nerve group block) OR (anesthesia OR anesthetic OR anaethesia OR analgesia) AND (hip OR hip joint) AND (arthroscopy)

## Part II: PAO/SHD Search Strategy for all Databases:

(((surgical hip dislocation[Title/Abstract]) OR (periacetabular osteotomy)) OR (hip PAO)) AND (((((((epidural block) OR (quadratus lumborum block)) OR (lumbar plexus block)) OR (anaesthesia)) OR (anesthetic)) OR (anesthesia)) OR (nerve block))
